# Editorial

**Published:** 1890-07

**Authors:** 


### Editorial.

### BLACKMAILING.

IT is a shame that we have no laws by which we can reach blackmailers properly.

There are so many people who stand ready to say "I told you so," that at the slightest hint or suspicion take the matter in hand, or, rather, in mouth, and ring the charges against any one's fair name that may be brought up.

Many a man's and woman's reputation has been ruined by these scandal-mongers, who were totally innocent of any crime.

The doctor is always considered fair game for the blackmailer, and suffers more than any other class, because of his position in society, which brings him in such intimate relation with the families.

We recall a half dozen cases in the past year where women, who had nothing to lose and all to gain, have brought the most damaging charges against some one of our profession, and where the doctor was proven absolutely innocent.

Why is it that blackmail is monopolized almost entirely by women? I think that in a large majority of cases a scheming man is really at the bottom of the trouble, and simply uses the woman as a cat's paw.

Would it not be a good idea to require a bond in all cases of damage suits, so that the defendant might have some redress? In this way many adventuresses would be foiled in their attempts to extort money from their would-be victims.

### DANGEROUS DRUGS.

Physicians, owing doubtless to constant use and familiarity with certain drugs, very frequently become careless and lose sight for the time being, of their dangerous character, and when death results, as it often does, it is regarded solely as the consequence of the disease under the treatment, and no fault is attributed to the remedy employed. This is true in relation to quinine, and eminently so in respect to salicin and the salicylates. Weber, Ringer, Murchison and a host of others equally competent, pronounce upon the dangerousness of drugs as a unit concerning the bad effects of this one. Within the past six years the writer has had the experience to see two cases, both middle-aged, rheumatic women, in which the poisonous action of the salicylate of soda was manifested. The cases had assumed the chronic stage with periodical exacerbations of fever and pain, and were treated according to the usual methods of employing this drug. After several weeks of its use there was excitement alternating with, as the medicine was withheld or pushed, lassitude, stupor, weakness of sight and hearing, then came on unsteady movements, hallucinations and delusions, pointing as was then thought, to the disease invading the meninges of the brain. Both of these cases gradually grew worse, and finally succumbed in dementia and coma. Literature contains several similar instances of the action of this salt in producing permanent mania,and we cannot be too careful in administering, watching its effects upon the body. Many deaths in rheumatism are attributed to the disease when doubtless the remedy is the most responsible.—B.

---

THE FIFTH ANNUAL BANQUET of the Atlanta Society of Medicine was a banquet indeed, and the Committee did themselves proud. It was given at E. F. Donehoo & Co's. Restaurant and served only as they can serve.

Judging from the empty plates, the M. D's. must have had a hard day's work.

This annual meeting is always given over to a flow of wit, and the wits displayed on this memorable occasion showed that none were too full for attendance, if we may judge from the remarks of Drs. Nicholson, Todd, Baird, Huzza, McRae, Howell, Avary, Duncan and others.

Drs. Nicolson, Cooper, Calhoun, Baird and Todd distinguished themselves as speech makers. Long may the Atlanta Society of Medicine live, and we are sure it will, if the enthusiasm that was shown at the Banquet is any criterion to judge by.

Our Dr. Calhoun has just had the degree of LL. D. conferred on him by the State University, and we are sure that the whole profession of the City feels as if it had been complimented; surely no one is more deserving of this high compliment than our Calhoun.

### DR. JAMES B. BAIRD.

The Southern Medical College has elected Dr. James B. Baird, of this City, to the Chair of Professor of Practice of Medicine. The college is certainly to be congratulated on securing the services of such an eminent practitioner. The students will have a treat in store next winter in hearing him lecture, as he is one of the finest lecturers in the South.

---

The ever progressive House of Parke, Davis & Co. are out this month with some seasonable suggestions as to eligible remedies for prevalent diseases of hot weather.

They have a very convenient list of intestinal sedatives, antiseptics, antispasmodics and anodynes for diarrhœal and dysenteric afflictions, some new expectorants of note for coughs and colds, and a normal liquid ipecac, always reliable as an emetic in cases of gastric disturbances due to accumulated fermented food so frequently a cause of infantile diarrhœa.

By way of gossip we may state that this house is largely increased in its facilities for the manufacture of pharmaceuticals. Buildings now in process of erection will double their capacity for production this year, and a new Laboratory, very complete in its appointments, is now being built for them in Canada.

---

### CORROSIVE SUBLIMATE FOR GRANULAR LIDS.

Dr. Arnautus has obtained excellent results in curing this troublesome disease by the application of solutions of corrosive sublimate of strength somewhat greater than is used in this country. He prescribes collyria or corrosive sublimate in the proportion of one to five hundred and one to four hundred, and of this one or two drops are instilled into the eye two or three times daily. He admits that solutions having this strength excite some transient irritation of the conjunctiva, but this disappears in the course of a few minutes, and may be prevented by the antecedent installation of a few drops of a solution of cocaine hydrochlorate. The remedy, he observes, costs little, and admits of easy application, and reabsorption of the granulations soon takes place. The effects of the solution are also well marked in causing the vascularization of the coma to disappear. The same treatment can be adopted in cases of ulcers of the cornea, many of which will rapidly heal under the influence of perchloride of mercury solution. Dr. Arnautus records cases showing the advantages to be derived from its use.—*Annales d' Oculistique.*

### THE WOMAN'S MEDICAL COLLEGE OF GEORGIA.

This institution begins its second annual term October 1st next, under the Presidency of Dr. A. G. Thomas, of this city. The class will consist exclusively of ladies. Half rates to wives and daughters of physicians, clergymen and Confederate veterans. For information, address J. W. Stone, M. D., box 215, Atlanta, Georgia.

### POISONING FROM SUBLIMATE DOUCHES.

M. Crequy reports a case of abortion with retention of the placenta (the os uteri being closed) in which he used vaginal douches of sublimate (1–2,000) three times a day for two days. The placenta was then discharged and a single intra-uterine douche with a double canula. Vaginal douches were then resumed as before, and at the end of two days the patient was seized with colic diarrhœa, vomiting and an intense stomatitis, causing sloughing of the cheeks. For several days the symptoms were grave, particularly that of anuria, which lasted three days, the patient finally making a good recovery. The writer adds that he has observed that sublimate disappears almost instantly from solutions when poured into metallic vessels, or when transferred by means of spoons, etc., from one vessel to another. It is, therefore, necessary to avoid the use of metallic irrigators, which retain the greater portion of the salt, allowing the water to escape almost pure, which, indeed, may possibly be one reason for the rarity of poisoning from sublimate solutions.—*La Sem. Med.*

"Pack my box with five dozen liquor jugs," contains all the letters in the alphabet.

